# PRAME Is a Golgi-Targeted Protein That Associates with the Elongin BC Complex and Is Upregulated by Interferon-Gamma and Bacterial PAMPs

**DOI:** 10.1371/journal.pone.0058052

**Published:** 2013-02-27

**Authors:** Frances R. Wadelin, Joel Fulton, Hilary M. Collins, Nikolaos Tertipis, Andrew Bottley, Keith A. Spriggs, Franco H. Falcone, David M. Heery

**Affiliations:** School of Pharmacy, Centre for Biomolecular Sciences, University of Nottingham, University Park, Nottingham, United Kingdom; New York University, United States of America

## Abstract

Preferentially expressed antigen in melanoma (PRAME) has been described as a cancer-testis antigen and is associated with leukaemias and solid tumours. Here we show that *PRAME* gene transcription in leukaemic cell lines is rapidly induced by exposure of cells to bacterial PAMPs (pathogen associated molecular patterns) in combination with type 2 interferon (IFNγ). Treatment of HL60 cells with lipopolysaccharide or peptidoglycan in combination with IFNγ resulted in a rapid and transient induction of *PRAME* transcription, and increased association of *PRAME* transcripts with polysomes. Moreover, treatment with PAMPs/IFNγ also modulated the subcellular localisation of PRAME proteins in HL60 and U937 cells, resulting in targeting of cytoplasmic PRAME to the Golgi. Affinity purification studies revealed that PRAME associates with Elongin B and Elongin C, components of Cullin E3 ubiquitin ligase complexes. This occurs via direct interaction of PRAME with Elongin C, and PRAME colocalises with Elongins in the Golgi after PAMP/IFNγ treatment. PRAME was also found to co-immunoprecipitate core histones, consistent with its partial localisation to the nucleus, and was found to bind directly to histone H3 *in vitro*. Thus, *PRAME* is upregulated by signalling pathways that are activated in response to infection/inflammation, and its product may have dual functions as a histone-binding protein, and in directing ubiquitylation of target proteins for processing in the Golgi.

## Introduction

PRAME/MAPE/OIP4 is an atypical cancer-testis antigen whose expression is associated with leukaemias and a large proportion of solid tumours (reviewed in [1]). Unlike other cancer-testis antigens whose expression is restricted to testis, the *PRAME* gene shows low level expression in other normal tissues including endometrium, ovary and placenta [2]. The restricted expression pattern of *PRAME* in normal tissues and its overexpression in tumours renders it a useful marker of minimal residual disease after chemotherapy, and an attractive target for immunotherapy, particularly in acute myeloid leukaemia (AML) and chronic myeloid leukaemia (CML) [2], [3], [4], [5], [6], [7], [8], [9], [10], [11], [12]. In *PRAME*-negative leukaemias the gene can be induced by demethylating agents [2], [13], [14], but little else is known regarding pathways that regulate *PRAME* expression.

*PRAME* is a member of a rapidly evolving multigene family, and encodes a leucine-rich repeat (LRR) protein sharing structural similarity with Toll-like receptors [1], [15]. The extensive duplication rate of *PRAME* and other cancer testis antigen genes is suggestive of roles in chemosensing, reproduction or immunity [15]. PRAME was originally identified in a yeast two-hybrid screen for proteins that bind outer membrane proteins of pathogenic bacteria [16], although until recently there has been little insight into its functions in mammalian cells. Nuclear localised PRAME has been implicated in transcriptional repression via association with retinoic acid receptor complexes [17]. More recently, while this study was in progress, PRAME was shown to be a chromatin-associated protein enriched at nuclear factor Y (NFY) target genes, in association with Elongin and Cullin-2 proteins [18]. However, a large proportion of endogenous PRAME protein is observed in the cytoplasmic compartment in different cell lines [1], [19]. Given its reported interaction with bacterial outer membrane proteins [16] and the rapid evolution of the *PRAME* multigene family in humans [15], similar to the Nacht, LRR, PYD domain (*NALP*) gene family [20], we hypothesised that *PRAME* might be regulated by signalling pathways activated in proinflammatory responses. We therefore set out to investigate whether *PRAME* expression is modulated by signalling molecules such as IFNγ or microbial PAMPs. We also endeavoured to isolate PRAME-interacting proteins, to provide further insight into its cellular functions.

## Results

### Upregulation of *PRAME* expression by IFNγ and bacterial PAMPs

**Figure 1:**
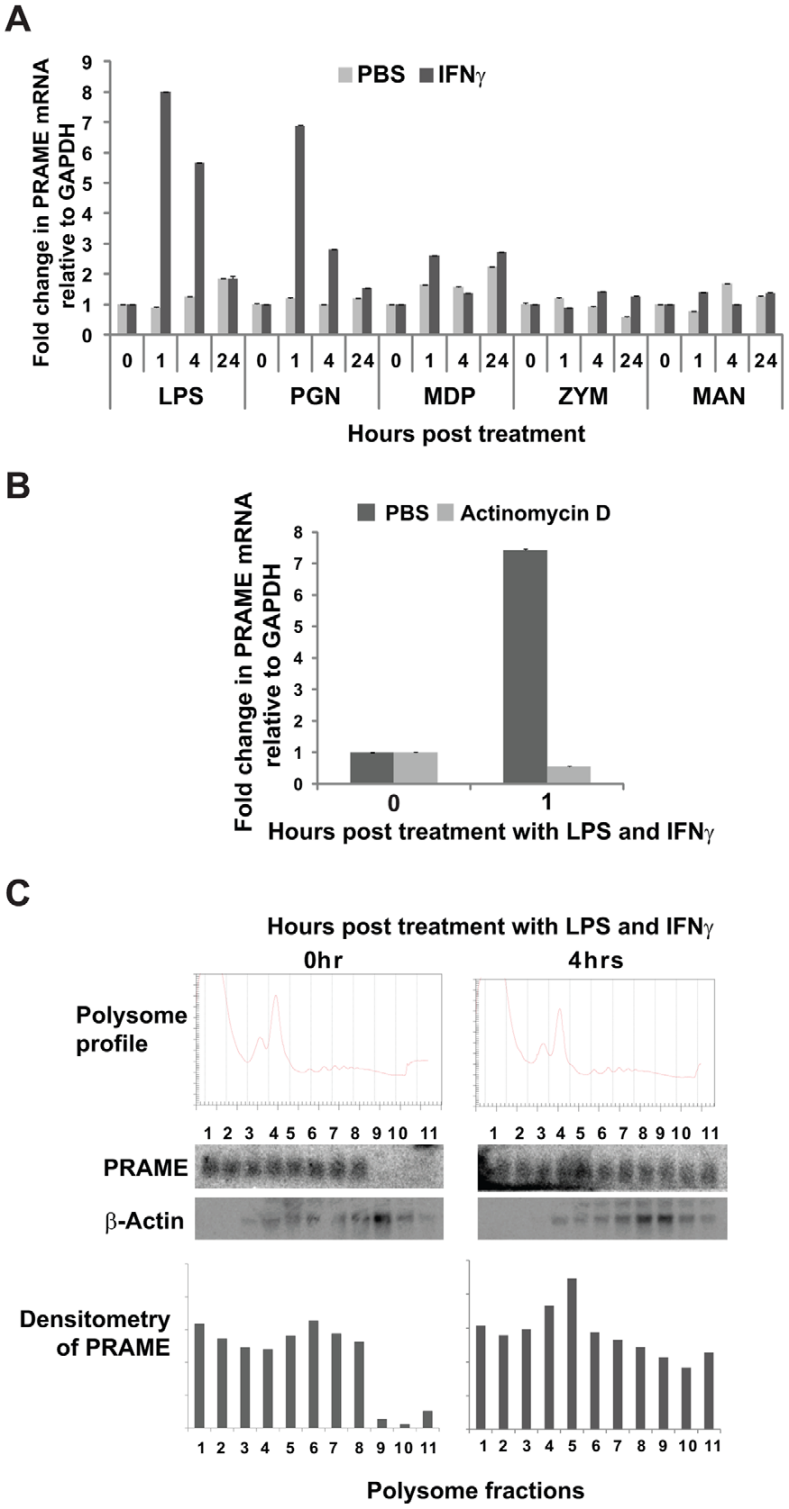
Transcriptional and translational regulation of *PRAME* by PAMPs/IFNγ. (**A**) RT-qPCR measurements of *PRAME* gene expression relative to control *GAPDH* in HL60 cells in response to treatment with different PAMPs including lipopolysaccharide (LPS), peptidoglycan (PGN), muramyl dipeptide (MDP), zymosan (ZYM) and mannan (MAN) either alone (PBS) or in combination with IFNγ. Numbers on the x-axis indicate the time in hrs post-treatment. qPCR quantifications were performed in triplicate and the data shown represents the mean of two independent experiments, with error bars indicating standard deviations. The data is presented as fold induction relative to levels obtained at 0 hr (baseline). (**B**) RT-qPCR experiment performed as in (A) showing effect of pre-treatment with actinomycin D (10 μg/ml) or PBS on induction of *PRAME* transcript in HL60 cells by LPS/IFNγ (1 hour). (**C**) Association of *PRAME* transcripts with polysomes in HL60 cells following treatment with LPS/IFNγ for 0 and 4 hrs. Following cycloheximide treatment and sucrose density centrifugation of HL60 cell lysates, gradients were fractionated with continuous monitoring at 254 nm, to generate polysome profiles (top panel). RNA extracted from polysome fractions was analysed by Northern blotting and the *PRAME* transcripts visualised by phosphoimager (middle panels) and quantified by densitometry (bottom panels). *β-actin* was used as a control probe.

PRAME protein contains a series of leucine-rich repeats similar to those found in the LRR protein family and is predicted to be structurally similar to human Toll-like receptors (TLRs) [1], [15]. Unlike the membrane-associated TLRs, PRAME is an intracellular protein found in both the nuclear and cytoplasmic compartments [1]. TLRs function in innate immunity as sensors of microbial PAMPs or other ligands, and their expression is regulated by these molecules and also by IFNγ, a proinflammatory cytokine produced in response to infection. We therefore assessed whether *PRAME* expression in leukaemic cells such as HL60 might be modulated by exposure to lipopolysaccharide (LPS) and IFNγ, either as single inducing agents or in combination. HL60 leukaemic cells have low levels of *PRAME* transcripts [1], and are known to express TLR2 and TLR4 [21]. Treatment of HL60 cells with IFNγ or LPS alone did not significantly alter the expression of *PRAME* (as determined by RT-qPCR – data not shown). However, as shown in Fig. 1A, a strong increase in *PRAME* transcript levels was observed within 1 hour of combined treatment with LPS and IFNγ. This increase was transient as *PRAME* levels were lower at 4 hours post-treatment, and restored to background levels within 24 hours, suggesting that turnover of *PRAME* mRNA may be relatively rapid. The induction of *PRAME* by LPS/IFNγ was inhibited by pre-treating cells with actinomycin D for one hour prior to addition of LPS/IFNγ (Fig. 1B), indicating that the increase in *PRAME* levels is due to *de novo* transcription rather than altered stability of the transcripts.

To examine whether LPS/IFNγ results in increased translation of *PRAME* transcripts, polysome gradients were prepared from extracts of HL60 cells treated or untreated with LPS and IFNγ. As shown in Fig. 1C, within 4 hours post-treatment with LPS/IFNγ, *PRAME* transcripts were substantially enriched in polysome fractions, as confirmed by densitometry (lower panels). In contrast, a control transcript (*β-actin*) showed little change. These results indicate that upregulation of *PRAME* gene expression by LPS/IFNγ in HL60 cells may be achieved not only through increased transcription, but also effects on translation of PRAME transcripts.

We also assessed whether *PRAME* expression could be induced by other microbial PAMPs in addition to LPS. As shown in Fig. 1A, treatment of HL60 cells with PAMPs such as muramyl dipeptide (MDP), zymosan A (ZYM) or mannan (MAN) for different times, either alone or in combination with IFNγ, had relatively little effect on *PRAME* expression. In contrast, peptidoglycan (PGN) in combination with IFNγ substantially increased *PRAME* expression, similar to the LPS/IFNγ response. Induction of *PRAME* by PGN/IFNγ was also transient, with peak levels observed within 1 hour post-treatment (Fig. 1A). Taken together, these results indicate that, similar to genes encoding other LRR proteins such as *NALPs* and *TLRs*, *PRAME* expression in HL60 cells may be rapidly induced by signalling pathways activated in response to bacterial infection/inflammation.

### Subcellular localisation of PRAME is altered by bacterial PAMPs/IFNγ

**Figure 2:**
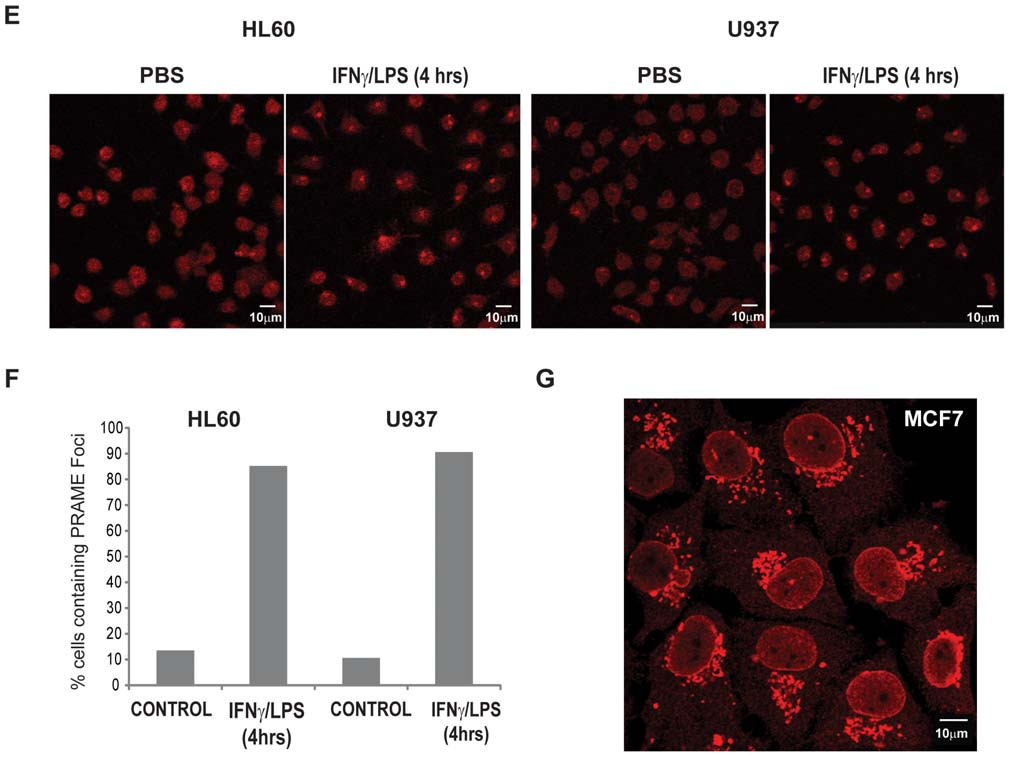
PRAME localises to the Golgi network following LPS/IFNγ treatment. (**A**) HEK293 cells (upper panels) were transiently transfected with PRAME-EGFP (green) and stained with α-PRAME antibody (red) to confirm the identity of the overexpressed EGFP fusion protein. U2OS cells (lower panels) were cotranfected with GFP (green) and PRAME-FLAG (red). Merged images indicate the extent of coincidence of the EGFP and α-PRAME signals, and nuclear DNA is indicated (blue). The right hand panels are western blots showing detection of GFP or PRAME-EGFP proteins in whole cell extracts of transfected U2OS cells. (**B**) Immunostaining of endogenous PRAME in HL60 cells using α-PRAME antibody following treatment with PBS, LPS/IFNγ or PGN/IFNγ for 4 hrs. (**C**) Immunostaining of endogenous PRAME in U937 cells with α-PRAME following treatment with LPS/IFNγ for 0, 1 and 4 hrs. (**D**) HL60 cells treated with LPS/IFNγ for 4 hrs and immunostained with α-Golgi 58K (green) and α-PRAME (red). Merged images show the extent of colocalisation of both proteins. For immunofluorescence (A–D), nuclear DNA was stained using Hoechst 33258 and images were captured using a LSM510 confocal laser scanning microscope. (**E**) Immunostaining of endogenous PRAME in HL60 cells using α-PRAME antibody following treatment with PBS or LPS/IFNγ for 4 hrs. (**F**) Quantification (n = 60) of the percentage of cells in (E) containing PRAME cytoplasmic foci in treated cells or controls. (**G**) Immunostaining of endogenous PRAME in MCF-7 cells using α-PRAME antibody.

Endogenous or recombinant PRAME proteins have been reported to localise to both nuclear and cytoplasmic compartments in a variety of cell types, including adherent cells [19] and leukaemic cell lines [1]. Transient transfection of U2OS or HEK293 cells with recombinant PRAME-EGFP or PRAME-FLAG revealed both nuclear and cytoplasmic staining, with the majority of signal (70–80% of total fluorescence) being observed in the cytoplasm of these cells within 24 hrs of transfection (Fig. 2A). Staining with a specific antibody recognising PRAME confirmed the identity of the recombinant PRAME-EGFP and PRAME-FLAG constructs, in contrast to neighbouring non-transfected cells which showed negligible endogenous levels of PRAME (Fig. 2A). In contrast to PRAME-EGFP, EGFP alone showed approximately equal distribution in the nucleus and cytoplasm whereas co-transfected PRAME-FLAG was predominantly cytoplasmic (Fig. 2A, lower panels). These observations are consistent with suggestions that PRAME is likely to have both nuclear and cytoplasmic functions, although the distribution between these compartments may vary in different cell types and conditions.

To determine whether exposure to bacterial PAMPs/IFNγ had any effect on the subcellular localisation of PRAME, we performed immunofluorescence staining of cells exposed to these agents, or controls. A longer treatment time (4 hours) was used to allow for expression of PRAME proteins in the cells. As shown in Fig. 2B, treatment of HL60 cells with LPS/IFNγ (middle panels) or PGN/IFNγ (lower panels) for 4 hours resulted in localisation of PRAME to large cytoplasmic foci. A similar effect was observed in the leukaemic cell line U937 in response to LPS/IFNγ treatment (Fig. 2C). Quantitation of PRAME-containing foci in LPS/IFNγ-treated and or control cells revealed a major change in the subcellular distribution of PRAME in response to LPS/IFNγ treatment (Fig. 2E&F).

The polarised distribution of the PRAME-containing foci, suggested that these structures may represent the *trans* Golgi network, and staining of HL60 cells with the 58K Golgi marker confirmed that these structures appear to be linked to the Golgi network and show considerable colocalisation with PRAME (Fig. 2D). We noted that the nuclei of LPS/IFNγ treated HL60 and U937 cells appeared to undergo a morphological change resulting in ‘kidney’ shaped nuclei being visible after 4 hours treatment (Fig. 2B&C), and that this phenotype was prevalent in many of the treated cells, but less obvious in controls (Fig. 2E). PRAME and the 58K Golgi marker accumulated at the apex of the invaginated region (Fig. 2D, lower panels). Thus, we conclude that in HL60 and U937 cells, cytoplasmic PRAME proteins are targeted to the Golgi network following exposure to LPS/IFNγ. Consistent with this, subcellular localisation prediction algorithms (PSORT.org) and other sequence analysis resources predict that PRAME is likely to be a Golgi-targeted protein [22]. Staining of endogenous PRAME in MCF-7 breast cancer cells also revealed its association with Golgi-like structures (Fig. 2G).

### Functional association of PRAME with Elongin BC complexes

**Figure 3:**
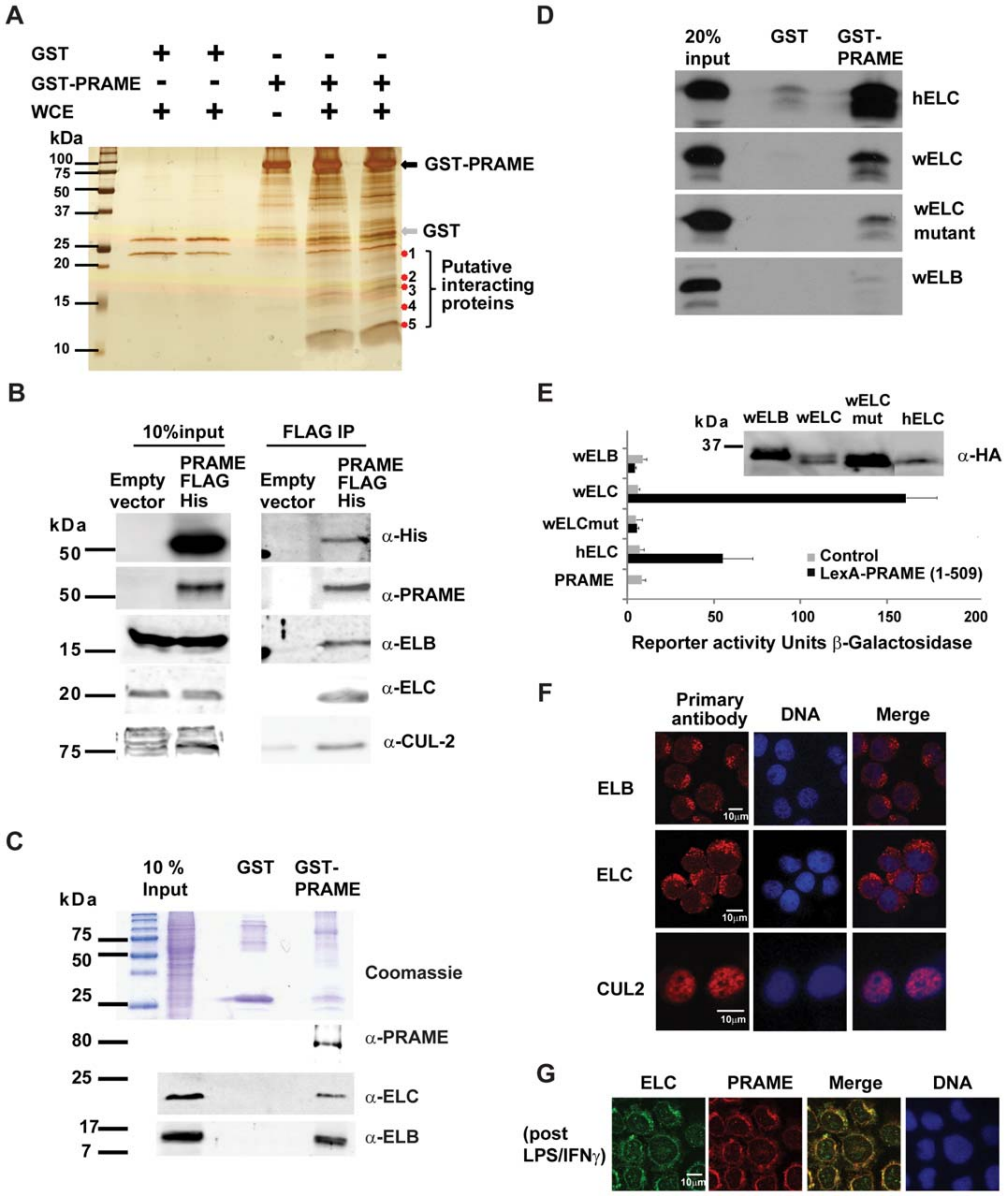
PRAME associates with the Elongin BC complex. (**A**) SDS-PAGE and silver staining showing affinity capture of proteins from HL60 whole cell extracts (WCE) by immobilised GST or GST-PRAME proteins. GST and GST-PRAME proteins are indicated. Putative PRAME-specific bands are indicated and bands of approximately 12 kDa and 17 kDa were excised for mass spectrometry analysis. (**B**) Co-immunoprecipitation of PRAME with Elongin complex components. Whole cell extracts of HEK293 cells transfected with PRAME-FLAG-6xHis (or empty vector control) applied to anti-FLAG sepharose beads as described in Materials and Methods. After extensive washing, co-purified PRAME and E3 ubiquitin ligase complex components were detected by western blotting using specific antibodies as indicated. (**C**) GST-pulldown experiment showing binding of ELB and ELC proteins in HL60 whole cell extracts to GST or GST-PRAME proteins. The top panel is a Coomassie-stained gel showing the input whole cell extract, and the purified GST and GST-PRAME proteins. The lower panels are western blots revealing PRAME, ELB and ELC proteins bound to GST proteins. (**D**) GST-pulldown experiments revealing interactions of ^35^ [S]-labelled *in vitro* translated human ELC (hELC), *C.elegans* ELC (wELC), *C.elegans* ELC (L47D-L49D-Y88D-Y91D) (wELC mutant) and *C.elegans* ELB proteins with GST or GST-PRAME. (**E**) Yeast two hybrid assays of LexA-PRAME interactions with GAL4 AD-fused human ELC (hELC) or *C.elegans* proteins (wELB, wELC, wELC mutant). Western blots of the HA-tagged elongin fusion proteins are also shown. Reporter activity is expressed as β-galactosidase activity normalised to amount of protein in the extracts. (**F**) Immunofluorescence staining showing subcellular localisation of endogenous ELC, ELB and CUL2 proteins in HL60 cells. (**G**) Immunofluorescence staining showing colocalisation of endogenous ELC and PRAME proteins in HL60 cells following treatment with LPS/IFNγ for 4 hours.

To further investigate the functions of PRAME, we performed affinity purification experiments to identify PRAME-associated proteins. Cleared whole cell lysates of HL60 cells were applied to GST-PRAME or control GST beads. After extensive washing, affinity purified proteins were visualised by silver staining, revealing a number of PRAME-specific bands (Fig. 3A). We selected two bands in the molecular range 10–20 kDa, which were among the most abundant and which were clearly absent in the controls (Fig. 3A). Following gel excision and extraction, these samples were subjected to reverse phase liquid-chromatography followed by high mass accuracy mass spectrometry. Polypeptides were identified using the MASCOT search engine [23]. As shown in Table 1, the top hits were the human ELB (elongin B) and ELC (elongin C) proteins. These are components of the E3 ubiquitin ligase complexes and are implicated in both protein ubiquitylation and transcription elongation.

**Table 1:** Mass Spectrometry Identification of PRAME Binding Proteins.

Band	Proteins identified	Number of peptides	Mowse score
1	ELB	6	359
	Histone H2B	2	73
2	ELC	6	397
	Histone H4	2	108

GST-PRAME binding proteins were affinity purified as described in Materials & Methods. Bands of approximately 17 kDa and 12 kDa (Fig. 3A) were excised for mass spectrometry analysis. MS/MS fragmentation data were used to search the human NCBI database using the MASCOT search engine. Probability-based Mowse scores are shown.

To validate these potential interactions, co-immunoprecipitation experiments were performed. As shown in Fig. 3B, endogenous ELB and ELC proteins were successfully co-purified from HEK293 cell extracts in complex with transiently expressed recombinant PRAME-FLAG-His. Similarly, GST-PRAME, but not GST alone, was able to purify both ELB and ELC from HL60 whole cell extracts (Fig. 3C). However, incubation of GST-PRAME with ^35^ [S]-labelled *in vitro* translated proteins revealed that direct interaction occurs between PRAME and ELC, but not ELB (Fig. 3D). This was confirmed using yeast two hybrid studies, which detected a strong interaction between LexA-PRAME and GAL4 activation domain-fused human or *C. elegans* ELC proteins, (which share 74% sequence identity), but not ELB (Fig. 3E). Moreover, this interaction with ELC was disrupted by mutations (L47D-L49D-Y88D-Y91D) in a conserved region required for binding of NLP48 [24] (Fig. 3E). No interaction was detected using ELB, although western blots on yeast cell-free extracts confirmed the expression of both ELB and ELC constructs (Fig. 3E). We also detected the Elongin BC-associated protein Cullin 2 (CUL2) in co-immunoprecipitation experiments using whole cell extracts from PRAME transfected HEK293 cells (Fig. 3B). Taken together, these results provide strong evidence for functional interaction between PRAME and components of the Elongin/Cullin E3 ubiquitin ligase complexes, consistent with the recent report from Costessi *et al* [18]. Our data further suggest that this is due to direct interaction of PRAME and ELC. Immunofluorescence staining of endogenous ELB and ELC proteins in HL60 cells revealed their localisation to cytoplasmic structures resembling Golgi (Fig. 3F). Unlike PRAME, this association of ELB and ELC with Golgi was not dependent on treatment with PAMP/IFNγ (Fig. 3F). Some nuclear staining of ELB and ELC was also detected, consistent with proposed roles of these proteins in transcriptional elongation [24]. Co-staining of LPS/IFNγ treated HL60 cells revealed extensive overlap of ELC and PRAME in Golgi-like structures, supporting the conclusion that these proteins colocalise to the Golgi (Fig. 3G). In contrast, CUL2 was found to be almost exclusively nuclear under similar conditions (Fig. 3F), which may be consistent with the identification of a CUL2/Elongin BC/PRAME complex in nuclear extracts [18]. Other ubiquitin ligase complexes have been implicated in trafficking to the Golgi and ER, and it remains possible that cytoplasmic PRAME can associate with other Cullins such as the Golgi-targeted CUL3 or CUL7 proteins [25], [26].

### Interaction of PRAME with histones

**Figure 4:**
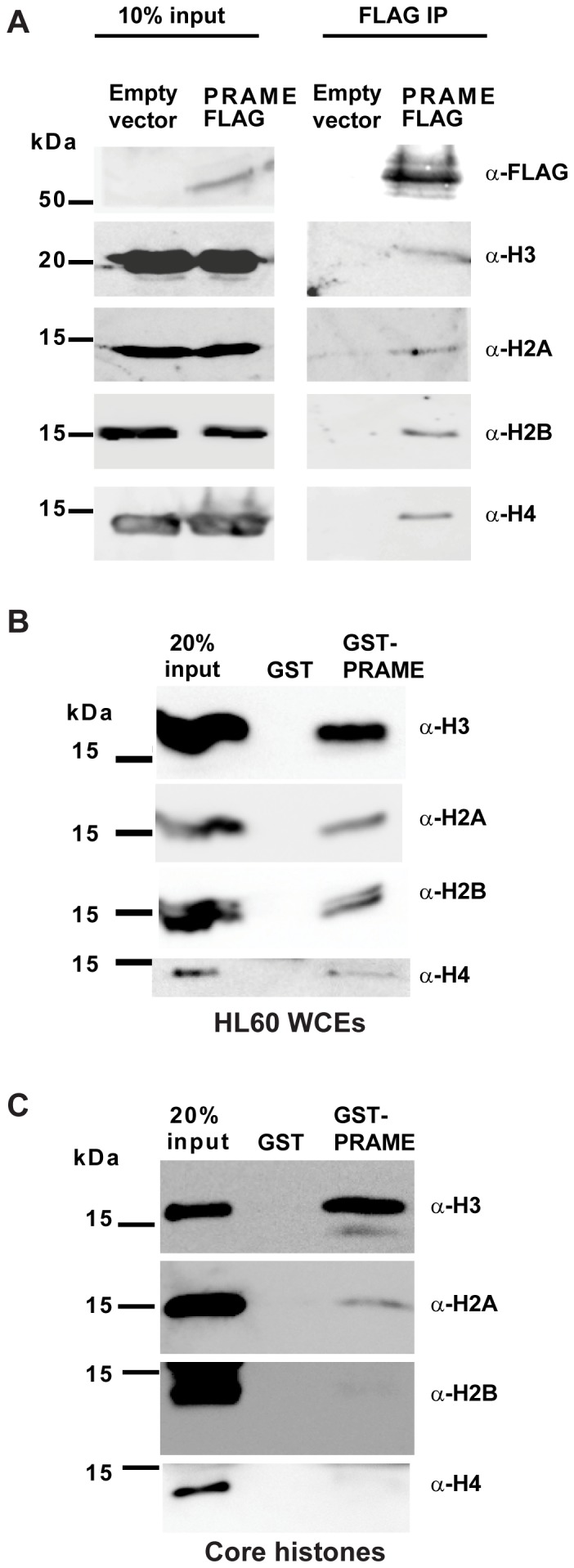
Binding of PRAME to histone H3. (**A**) Co-immunoprecipitation of endogenous histones with PRAME-FLAG-6xHis isolated from extracts of transfected HEK293 cells. Input lanes (left panels) show the presence of endogenous or FLAG-tagged proteins in extracts from cells transfected with PRAME-FLAG or empty vector. After IP with anti-FLAG antibody, immunoblots were performed with the antibodies indicated. (**B**) GST-pulldown experiment showing association of histones with GST or GST-PRAME proteins. Whole cell extracts of HL60 cells were incubated with immobilised GST or GST-PRAME proteins, and bound proteins separated by SDS-PAGE. Immunoblots were performed with specific antibodies to detect association of histones H2A, H2B, H3 and H4 with GST proteins. (**C**) Direct association of histones with GST or GST-PRAME proteins. Core histone preparations were incubated with GST beads. Following extensive washing, bound histones were separated by SDS-PAGE and revealed by western blotting using specific antibodies as indicated.

In addition to Elongins, the mass spectrometry analysis also detected histones as putative PRAME binding proteins (Table 1). Nuclear localised PRAME was recently reported to act as a coregulator of gene expression, being recruited to a subset of NFY-regulated promoters [18]. While PRAME was demonstrated to associate with chromatin, it was reported not to interact with DNA directly, thus the mechanism by which it associates with chromatin was not established [18]. Immunoprecipitation of epitope-tagged PRAME from HEK293 whole cell extracts confirmed that all four core histones can co-immunoprecipitate with PRAME (Fig. 4A). In addition, GST-pulldown experiments using HL60 whole cell extracts (Fig. 4B) or core histone preparations (Fig. 4C) also detected interactions of PRAME with histone H3. Whole cell extracts may contain histones as nucleosomes, which could account for the co-precipitation of all four core histones from cell extracts. However core histone preparations are generally DNA-free. Therefore, the strong detection of histone H3 in core histone binding experiments (Fig. 4C) suggests that direct interaction of PRAME with H3 may enable PRAME to associate with chromatin.

## Discussion

Although *PRAME* is an important biomarker in certain haematological malignancies and solid tumours, the regulation of this gene is poorly understood. Expression of *PRAME* in malignant cells is known to be subject to epigenetic regulation, as we and others have shown that DNA demethylating agents can induce *PRAME* in tumours and leukemic cell lines [2], [13], [14]. However, until now, little else was known regarding the regulation of *PRAME* expression. In this study we provide the first evidence that *PRAME* may be regulated by signalling pathways involved in innate immune responses.

Innate immune pathways coordinate appropriate responses to infection or immunological challenge, and also play a prominent role in the regulation of haematopoiesis [27], [28]. Aberrant activity of such pathways is associated with myelodysplastic syndromes and haematological malignancies [28]. For example, chronic inflammation is involved in the origin and progression of certain B-cell lymphomas and can be mediated through TLRs and other components of the innate immune system, that are activated through recognition of microbial PAMPs [29]. Similar pathways are likely to be involved in progression of myeloid leukaemias. For example, TLR2 and TLR4 which mediate responses to LPS and PGN are both activated and upregulated following exposure of HL60 cells to these agents [21]. Similarly, PGN-mediated activation of TLR2 and other pathways has been shown to enhance expression of chemoattractant genes in human monocytic leukaemia cells [30]. TLR signalling converges with cytokine signalling and other pathways to amplify or moderate immune responses. For example, the proinflammatory cytokine IFNγ is produced by macrophages following intracellular invasion and is known to prime macrophages to respond to infection [27]. This combination of IFNγ with TLR activation can ensure rapid induction of subsets of proinflammatory genes. Thus, given the predicted structural similarity of PRAME to the extracellular LRR domains of TLR2 and TLR4 [1], [15], and its reported ability to bind bacterial OPA proteins [16] we hypothesised that PRAME might have some role as an intracellular sensor of invading pathogens. Such an inducible expression profile might account for its generally low or undetectable levels in normal tissues. These observations prompted us to investigate whether *PRAME* expression might be upregulated in response to microbial PAMPs and/or IFNγ.

Solid tumours can also induce proinflammatory pathways, producing a microenvironment that favours tumour growth and proliferation. In addition, tumour-associated leucocytes may produce cytokines that stimulate tumour growth and angiogenesis [31]. This can occur through activation of TLRs, or through IFNγ production resulting in activation of downstream effectors such as nuclear factor kappa B (NFκB), interferon regulatory transcription factors (IRFs) and signal transducers and activators of transcription (STATs) [31]. These transcription factors are implicated not only in the regulation of proinflammatory genes, but also in cancer-related inflammation and in aberrant gene regulation in tumours [31]. The mechanism by which PAMP/IFNγ treatment induces *PRAME* transcription remains to be characterised, but is likely to involve one or more of these effectors. Interestingly, the proximal promoter of the *PRAME* gene contains a number of potential binding sites for NFκB, IRFs and STATs. Further studies will be required to characterise the *cis* and *trans* acting factors that regulate *PRAME* expression.

In addition to the observed effects on *de novo* transcription (Fig. 1A&B), treatment of HL60 cells with PAMPs/IFNγ also appeared to increase recruitment of *PRAME* transcripts into polysomes (Fig. 1C). This suggests that *PRAME* may also be subject to translational regulation, perhaps as a consequence of the extended 5′UTR sequences present in *PRAME* transcripts [1]. It remains to be seen whether such insights into the regulation of this tumour biomarker will enable the manipulation of its expression in *PRAME*-negative tumours. As *PRAME* is undergoing evaluation in clinical trials as an anti-cancer vaccine target in non-small cell lung cancer (NCT01159964), its re-activation in tumours might facilitate their targeting by immunotherapeutics.

Endogenous and overexpressed PRAME proteins were observed in both the nucleus and cytoplasmic compartments in different cancer cell lines (Fig. 2A–D). In addition to the observed upregulation of PRAME expression, treatment of HL60 or U937 leukemic cells with PAMPs/IFNγ resulted in the accumulation of cytoplasmic PRAME in Golgi-like structures (Fig. 2B&C). This was supported by the observed colocalisation of PRAME and the 58K Golgi marker (Fig. 2D). A significant proportion of treated HL60 cells were found to begin to undergo lobulation of the nucleus (Fig. 2E). Similar effects on nuclear shape have been reported for a HL60 cell line undergoing retinoic acid induced granulopoiesis, with the Golgi juxtapositioned between nuclear lobes [32]. Interestingly, nuclear lobulation in macrophages enables them to infiltrate other tissues in response to chemoattractants. Consistent with reports that PRAME is expressed in breast tumours, [33], [34] PRAME was readily detected in MCF7 cells, by immunofluorescence staining (Fig. 2G), where it also showed a strong association with Golgi-like structures. The functional consequences of the association of PRAME with the Golgi remains to be determined, such as whether PRAME plays role in nuclear lobulation in macrophages or other monocytic cells.

Due to poor performance of available PRAME antibodies in detecting endogenous proteins by western blotting, we were unable to reliably detect an increase in intracellular levels of soluble PRAME in HL60 cells following IFN/LPS induction. It is possible that the detection of endogenous PRAME in cell-free extracts might also be hampered by its retention in the insoluble fraction of cell lysates, due to increased localisation to Golgi/ER macromolecular structures.

Using an affinity purification approach to explore the functions of PRAME through its molecular partners, we identified an interaction of PRAME with the Elongin/Cullin E3 ubiquitin ligase complex as confirmed in a range of binding assays and co-purification experiments (Fig. 3A–G). Co-immunoprecipitation experiments detected ELB, ELC and CUL2 proteins in complex with PRAME in HL60 cells. This association of PRAME with the Elongin/Cullin complex is consistent with a recent report from Costessi *et al*. [18], in which a similar complex was identified in K562 cells, which contain high levels of PRAME protein. Interestingly, these data are also consistent with a previous study which noted an interaction between PRAME and the E2 ubiquitin enzyme UBE2F, in a yeast two-hybrid screen [35]. In our hands, direct interactions appear to occur between PRAME and ELC, based on yeast two-hybrid and GST-pulldown assays (Fig. 3D&E), which is consistent with observations for other complexes involving LRR and BC box proteins. Thus, as observed for its counterparts in similar complexes, the function of PRAME may be to target substrates for ubiquitylation by the Elongin/Cullin complex, although the identity of these potential substrates remains unknown.

Elongins have both nuclear and cytoplasmic functions, having roles in protein ubiquitylation and gene transcription [24]. Interestingly, staining of HL60 cells for endogenous ELC revealed it to be mainly cytoplasmic and at least partly associated with the Golgi network (Fig. 3F&G), thus indicating that a major site of interaction of PRAME and Elongins is in the cytoplasmic compartment. CUL2 on the other hand appeared to be almost exclusively nuclear (Fig. 3F). We did not observe any major change in the subcellular localisation of ELB, ELC or Cul2 following IFN/LPS treatments (data not shown), and antibody conflicts precluded co-staining of ELC with the Golgi marker. However, the localisation of ELB and ELC to polarised cytoplasmic foci is consistent with Golgi/ER association. It is not yet known whether PRAME can associate with other Cullin complexes, such as Golgi-resident CUL3 or CUL7 proteins [25], [26].

Our affinity purification experiment also identified a novel interaction of PRAME with core histones (Table 1). Direct interaction appears to be mediated chiefly through histone H3, which shows strongest association with PRAME (Fig. 4C). This result is consistent with the recent report that nuclear PRAME can be recruited to chromatin and functions in gene regulation, although it lacks DNA binding activity [18]. In addition, PRAME has previously been reported to co-immunoprecipitate with the histone methyltransferase EZH2 [17], which promotes trimethylation of H3K27. Given its interaction with Elongin BC complexes, it is therefore conceivable that PRAME might play some role in histone ubiquitylation, although there is currently no evidence to support this. Several different nuclear E3 ubiquitin ligase complexes are known to catalyse the addition of ubiquitin to core histones [36]. This includes the CUL2 complex which promotes monoubiquitylation of H2B at residue K120, through the interaction of the histone with the BC-box protein BAF250b [37]. Future studies will therefore need to focus on assessing whether depletion or overexpression of PRAME can influence ubiquitylation of histones or other target proteins.

**Figure 5:**
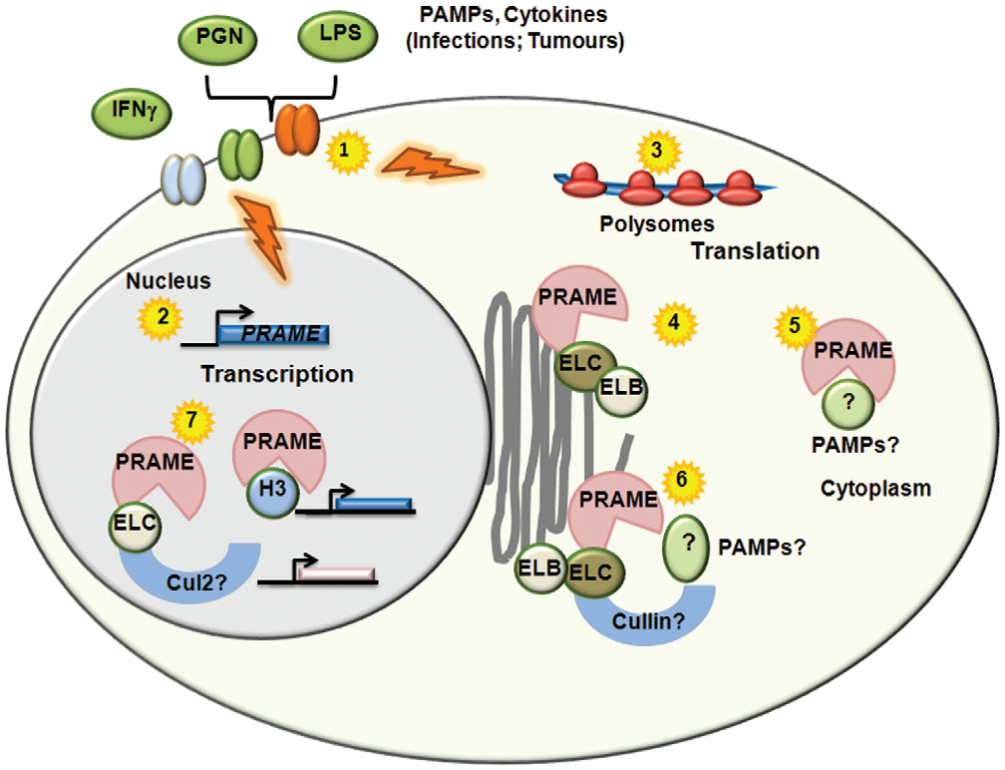
Potential Functions and Regulation of PRAME. Activation of TLRs and other signalling receptors by PAMPs and cytokines associated with infections or tumours (1) results in both transcriptional (2) and translational (3) upregulation of *PRAME*. Cytoplasmic PRAME can associate with Elongin proteins located in the Golgi/ER network (4). Association of PRAME with intracellular PAMPs or other molecules (5) may facilitate their targeting to the Golgi for modification, secretion or destruction by Elongin/Cullin Ubiquitin ligases (6). Interactions of PRAME proteins with Elongins, Cul2 or Histone H3 in the nucleus (7) may be involved in the regulation of gene expression.

In conclusion, our study provides novel insights into the functions and regulation of PRAME, as summarised in Figure 5. We hypothesise that, like TLRs, PRAME may be upregulated in response to encounters with microbial pathogens, and may be involved in targeting intracellular PAMPs to the Golgi for ubiquitylation and processing. The activation of similar cytokine signalling pathways in cancer cells might therefore account for the expression of PRAME in tumours, and its low levels in normal tissues. Finally, direct interactions of PRAME with histone H3 in addition to elongins may be important in its functions in gene transcription. These insights will aid future efforts to establish whether PRAME expression in tumours has roles in the initiation or progression of haematological malignancies, and/or in immune responses to infection.

## Materials and Methods

### Cell lines, reagents and transient transfections

HL60, U937, U2OS, MCF-7 and HEK293 cell lines were purchased from the European Collection of Cell Cultures (ECACC). HL60 and U937 leukaemic cell lines were maintained in RPMI-1640. HEK293, U2OS and MCF-7 cells were maintained in Dulbecco's modified Eagle medium. Culture medium was supplemented with 10% foetal bovine serum, 2 mM glutamine, 100units/ml penicillin and 100 µg/ml streptomycin and cells were maintained at 37°C in a 5% CO_2_ incubator. Cell cultures were split 24 hrs prior to treatment with 100U/ml interferon-gamma (IFNγ, AbD Serotec), 50 ng/ml *Salmonella minnesota* lipopolysaccharide (LPS, Sigma-Aldrich), 5 μg/ml *Bacillus subtilis* peptidoglycan (PGN, Sigma-Aldrich), 10 μg/ml muramyl dipeptide (MDP, Sigma-Aldrich), 100 μg/ml zymosan A (ZYM, Sigma-Aldrich) or 10 μg/ml mannan (MAN, Sigma-Aldrich). Actinomycin D (10 μg/ml, Sigma-Aldrich) was applied to cells 1 hr prior to other treatments.

U2OS and HEK293 cells were seeded onto coverslips 16 hrs prior to transient transfection using CalPhos^TM^ mammalian transfection kit (Clontech).

### Expression plasmids

PRAME-EGFP and PRAME-FLAG expression vectors [19] were gifts from P.Gailly. Yeast two hybrid pGADT7 vectors expressing *C.elegans* CUL2, ELB, ELC, ELC mutant (L47D/L49D/Y88D/Y91D) and human ELC [38] proteins containing haemagluttinin (HA) epitope-tags were gifts from K.Yamanaka. A PCR fragment containing *PRAME* coding sequence was generated from pCMV-Sport6-PRAME (Geneservice) using the following primers: 5′-atggaacgaaggcgtttg-3′ and 5′-ctagttaggcatgaaacagggg-3′. The fragment was subcloned into *SmaI* digested pBSKSII, to generate pBSKSII-PRAME. pBSKSII-PRAME was digested with *BamHI* and *XhoI* and the insert fragment was subcloned into pGEX4T1, to generate GST-PRAME. This fragment was also subcloned into pBTM116mod and pASV3mod [35] to generate LexA-PRAME and VP16-PRAME, respectively. PRAME-FLAG-His was generated by PCR from PRAME-FLAG [19] using primers: 5′-aaaaggatccgccgccatggaacgaaggcgttt g-3′ and 5′-aaaactcgagctaatggtgatggtgatgatgcttgtcatcgtcatccttgtagta-3′. The insert was subcloned into the *BamHI* and *XhoI* sites in pcDNA3.1(+).

### Polysome analysis and Northern blotting

After cycloheximide treatment of HL60 cells and preparation of cell lysates [39], sucrose density gradients were used to separate ribosomes into polysomal and subpolysomal complexes. Gradients were fractionated with continuous monitoring at 254 nm, and RNA was isolated from each fraction as described previously [40]. Northern analysis of RNA isolated from sucrose density gradients was performed as described previously [41], using ^32^P-labelled probes for full-length PRAME and actin. Activity was visualised using a Storm825 phosphorimager (GE Healthcare) and ImageQuantTL software (GE Healthcare).

### Reverse transcription PCR and qPCR

RNA was extracted using EZNA^TM^ total RNA kit I (Omega Bio-Tek). RNA was reverse transcribed using oligo(dT)_12-18_ and Superscript III (Invitrogen) according to the manufacturer's instructions. RTqPCR was performed using Brilliant II SYBR green qPCR mix (Stratagene) and primers: PRAME: 5′-tgctgatgaagggacaac at-3′ and 5′-cag cacttgaagtttccacct-3′; GAPDH: 5′-aggtgaaggtcggagtcaac-3′ and 5′-gatgacaagcttcccgttct-3′. PCR was conducted using Mx3005P real-time PCR system (Stratagene) with the following conditions: hot start activation at 95°C for 10 mins followed by 40 cycles of 95°C for 30 secs, 59°C for 1 min. PCR reactions were performed in triplicate for each sample. The threshold cycle number of PRAME was normalised to that of GAPDH. Fold increase was calculated using the comparative (ΔΔCt) method, as previously described [42].

### Western blotting, antibodies and immunofluorescence staining

Proteins were resolved on standard SDS-PAGE gels and transferred to nitrocellulose membranes. Membranes were preblocked in 3% non-fat milk in PBS prior to addition of primary antibodies. For Western blotting, coimmunoprecipitations, and immunofluorescence, the following antibodies were used: anti-PRAME (ab32185, Abcam WB 1∶500, IF 1∶100), anti-FLAG M2 (F3165, Sigma-Aldrich WB 1∶500, IF 1∶100), anti-Golgi 58K (ab27043, Abcam IF 1∶100), anti-His (05–531, Millipore WB 1∶1000), anti-ELB (sc11447, Santa Cruz Biotechnologies WB 1∶200, IF 1∶100), anti-ELC (sc1559, Santa Cruz Biotechnologies WB 1∶500, IF 1∶10), anti-CUL2 (ab1870, Abcam WB 1∶500, IF 1∶250), anti-HA (3F10, Roche WB 1∶500), anti-H2A (ab18255, Abcam WB 1∶500), anti-H2B (ab1790, Abcam WB 1∶500), anti-H3 (ab1791, Abcam WB 1∶1000), anti-H4 (ab7311, Abcam WB 1∶500), anti-GST (Ab-2, Oncogene Research Products WB 1∶500), anti-GFP (ab6556 Abcam WB 1∶500). The following horse radish peroxidise-linked secondary antibodies were used for Western blotting and co-immunoprecipitations at 1∶5000: chicken anti-mouse (sc2954, Santa Cruz Biotechnologies), donkey anti-goat (30220–210, Alpha Diagnostics International), goat anti-rabbit (sc2004, Santa Cruz Biotechnologies). For immunofluorescence, the following secondary antibodies were used at 1∶500: alexa fluor chicken anti-mouse 488 (A21200), alexa fluor chicken anti-goat 594 (A21468) and alexa fluor chicken anti-rabbit 594 (A21442) Invitrogen. Y2H fusion proteins in yeast cell-free extracts were detected using anti-LexA (06–719, Millipore WB 1∶1000) or anti-HA (3F10, Roche WB 1∶500).

Immunofluorescence staining and image capture was performed using a Leica LSM510 confocal microscope essentially as described previously [39]. U2OS, HEK293 and MCF-7 cells were grown on glass coverslips, while non-adherent cells were mounted on glass slides at 1000rpm for 5 minutes using a Cytospin 4 cytocentrifuge. Representative images of IFNγ/PAMP-treated cells and PBS-treated controls immunostained with α-PRAME antibody were quantified by manual scoring of the percentage of cells (n = 60) showing large cytoplasmic PRAME-containing foci.

### Protein affinity purification, co-immunoprecipitation and GST-pulldown assays

Preparation of whole cell extracts of mammalian cells was performed by using freeze thaw cycles in cell lysis buffer with protease inhibitors as described previously [39]. GST-PRAME and empty pGEX-4T1 (or GST) vectors were transformed into *E.coli Rosetta* cells and selected on LB plates containing ampicillin. Single colonies were cultured to exponential phase in LB ampicillin broth at 37°C, and GST protein expression was induced by addition of, 0.5 mM isopropyl β-D-1-thiogalactopyranoside (Merck) for 16 hrs at 20°C. Bacterial pellets were resuspended in NTN (20 mM Tris pH8, 100 mM NaCl, 0.5% NP40, 1 mM DTT 1X Complete EDTA-free protease inhibitor cocktail (Roche 11836170001), sonicated and then centrifuged at 15000rpm for 40 mins at 4°C. The supernatant was added to prewashed glutathione sepharose beads (GE Healthcare) that had been blocked with 0.5% milk and incubated for 16 hrs at 4°C with rotation. The beads were washed extensively in NTN buffer. Affinity capture was performed using 1 mg whole cell extract or 15 μg purified core histones (Sigma H9250). GST, GST-PRAME and other proteins bound to beads were resolved by SDS-PAGE and revealed by western blotting using the antibodies described above.

### Silver staining and mass spectrometry

After protein separation by SDS-PAGE, gels were fixed with 2 washes in 40% methanol/10% acetic acid, sensitized in 30% methanol, 0.2% sodium thiosulphate, 6.8% sodium acetate, impregnated with 0.25% silver nitrate and developed in 2.5% sodium carbonate, 0.15% formaldehyde, 0.003% sodium thiosulphate. The staining reaction was stopped in 1.46% EDTA.

Bands of interest were excised and analysed by the Cambridge Centre for Proteomics. This involved overnight trypsin digestion of the proteins in the gel bands followed by reverse phase liquid-chromatography and then high mass accuracy mass spectrometry. For identification of proteins, the MS/MS fragmentation data were used to search the human National Centre Biological Information database using the MASCOT search engine. Probability-based Mowse scores were used for evaluating peptide identifications [43]. Only those peptide matches with a Mowse score >38 were considered significant and reported.

### Yeast-two hybrid assays

Yeast transformations, yeast two-hybrid reporter assays, yeast cell-free extract preparations for western blotting were performed essentially as described previously [35].
