# Correction: PRAME Is a Golgi-Targeted Protein That Associates with the Elongin BC Complex and Is Upregulated by Interferon-Gamma and Bacterial PAMPs

**DOI:** 10.1371/journal.pone.0129297

**Published:** 2015-06-12

**Authors:** Frances R. Wadelin, Joel Fulton, Hilary M. Collins, Nikolaos Tertipis, Andrew Bottley, Keith A. Spriggs, Franco H. Falcone, David M. Heery

Fig 2 is incorrect. Panels A-D are inadvertently missing. The authors have provided a corrected version here.

**Fig 2:**
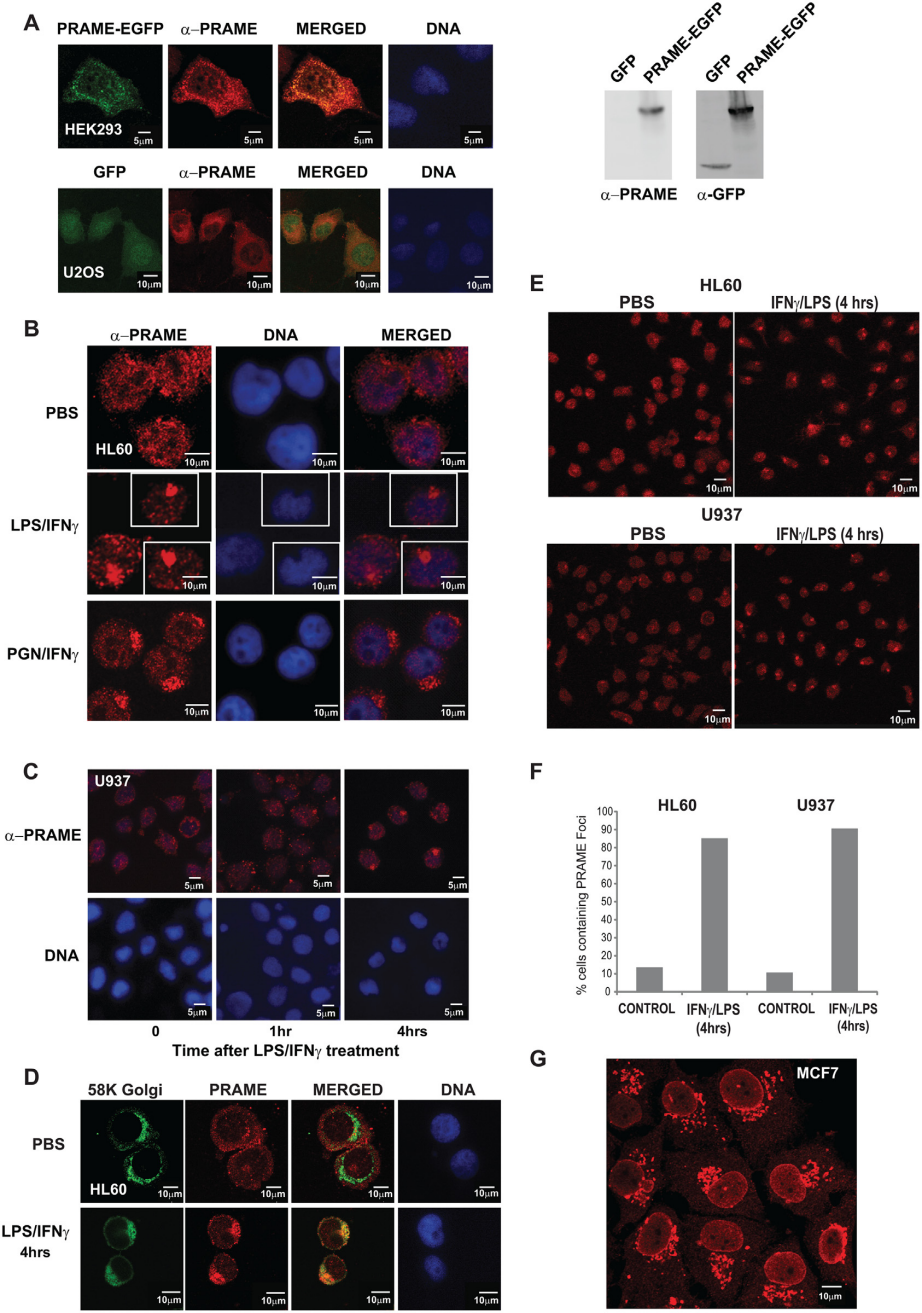
PRAME localises to the Golgi network following LPS/IFNγ treatment. (**A**) HEK293 cells (upper panels) were transiently transfected with PRAME-EGFP (green) and stained with α-PRAME antibody (red) to confirm the identity of the overexpressed EGFP fusion protein. U2OS cells (lower panels) were cotranfected with GFP (green) and PRAME-FLAG (red). Merged images indicate the extent of coincidence of the EGFP and α-PRAME signals, and nuclear DNA is indicated (blue). The right hand panels are western blots showing detection of GFP or PRAME-EGFP proteins in whole cell extracts of transfected U2OS cells. (**B**) Immunostaining of endogenous PRAME in HL60 cells using α-PRAME antibody following treatment with PBS, LPS/IFNγ or PGN/IFNγ for 4 hrs. (**C**) Immunostaining of endogenous PRAME in U937 cells with α-PRAME following treatment with LPS/IFNγ for 0, 1 and 4 hrs. (**D**) HL60 cells treated with LPS/IFNγ for 4 hrs and immunostained with α-Golgi 58K (green) and α-PRAME (red). Merged images show the extent of colocalisation of both proteins. For immunofluorescence (A–D), nuclear DNA was stained using Hoechst 33258 and images were captured using a LSM510 confocal laser scanning microscope. (**E**) Immunostaining of endogenous PRAME in HL60 cells using α-PRAME antibody following treatment with PBS or LPS/IFNγ for 4 hrs. (**F**) Quantification (n = 60) of the percentage of cells in (E) containing PRAME cytoplasmic foci in treated cells or controls. (**G**) Immunostaining of endogenous PRAME in MCF-7 cells using α-PRAME antibody.

## References

[1] Wadelin FR, Fulton J, Collins HM, Tertipis N, Bottley A, Spriggs KA, et al (2013) PRAME Is a Golgi-Targeted Protein That Associates with the Elongin BC Complex and Is Upregulated by Interferon-Gamma and Bacterial PAMPs. PLoS ONE 8(2): e58052 doi: 10.1371/journal.pone.0058052 23460923 10.1371/journal.pone.0058052 PMC3584020

